# New insights in fluid monitoring for surgical patients. A concept study

**DOI:** 10.3389/fmedt.2025.1619238

**Published:** 2025-07-21

**Authors:** Audrius Andrijauskas, Povilas Andrijauskas, Darius Dilijonas, Tomas Jovaiša, Vaidotas Marozas, Edgaras Stankevičius, Axel Kerroum, Darius Čincikas, Saulė Švedienė, Giedrius Kvederas, Narūnas Porvaneckas, Christer Svensen

**Affiliations:** ^1^ Clinic of Anesthesiology and Intensive Care, Institute of Clinical Medicine, Faculty of Medicine, Vilnius University, Vilnius, Lithuania; ^2^ II Department of Anesthesiology and Intensive Care, Vilnius University Hospital Santaros Klinikos, Vilnius, Lithuania; ^3^ Institute of Social Sciences and Applied Informatics, Kaunas Faculty, Vilnius University, Kaunas, Lithuania; ^4^ Faculty of Electrical and Electronics Engineering, Kaunas University of Technology, Kaunas, Lithuania; ^5^ Biomedical Engineering Institute, Kaunas University of Technology, Kaunas, Lithuania; ^6^ Institute of Physiology and Pharmacology, Lithuanian University of Health Sciences, Kaunas, Lithuania; ^7^ Department of Anesthesiology, Lausanne University Hospital, Lausanne, Switzerland; ^8^ Clinic of Rheumatology, Traumatology Orthopaedics and Reconstructive Surgery, Institute of ClinicalMedicine of the Faculty of Medicine, Vilnius University, Vilnius, Lithuania; ^9^ Karolinska Institutet, Department of Science and Ediucation, Section of Anesthesia and Intensive Care, Södersjukhuset, Stockholm, Sweden

**Keywords:** perioperative fluid therapy, hemoglobin, hematocrit, hemodilution, plasma dilution, hydration, transcapillary reflux, innovative technique

## Abstract

**Purpose:**

This study evaluates the primary hypothesis of red cell mass (RCM) dependent amplitude of homeostatically acceptable limits of fluctuation in plasma dilution by exploring the correlation between RCM-specific equilibrated hematocrit (EQ_Hct) and amplitude of plasma dilution during perioperative mini Volume Loading Test (mVLT).

**Materials and methods:**

We retrospectively analyzed data from our previous RCTs, including 1,651 invasive arterial plasma dilution (aPD), 1,645 noninvasive “capillary” plasma dilution (cPD) and 236 estimates of EQ_Hct from 236 perioperative mVLT sessions. The cPD was estimated using noninvasive hemoglobin (SpHb, Masimo Radical 7, Irvine, CA) measurement. Fixed number of crystalloid boluses was used in 36 and 48 elective total knee arthroplasty (TKA) patients, and individualized number of boluses in 34 total hip arthroplasty (THA) patients for whom the number of boluses depended on the advices by our prototype automated clinical decision support system (ACDSS).

**Results:**

The primary hypothesis was confirmed—aPD decreased as EQ_Hct decreased when EQ_Hct <40%, and a very weak positive correlation was found between EQ_Hct and absolute aPD (Spearman's correlation coefficient 0.1025, *p* < 0.001). It was also confirmed when non-invasive data sets were used. A very weak negative correlation between HctEQ values and absolute cPD values (Spearman's correlation coefficient 0.0640, *p* *=* 0.0149).

**Conclusion:**

This study points to the feasibility of Photoplethysmography (PPG) based estimates of hemoglobin concentration for continuous noninvasive monitoring of fluid accumulation and detecting imminent edema using the Homeostatic Blood States (HBS) theory and transcapillary reflux model. The ACDSS-guided fluid loading has a potential to minimise unnecessary fluid accumulation. Further research is needed to explore and improve these techniques.

## 1 Introduction

Intravenous administration of fluids plays a fundamental role in the treatment of critically ill and patients going through major surgery. Both very high and very low perioperative fluid volumes have been shown associated with an increase in complications after noncardiac surgery (1). Fluid therapy in cardiac surgery patients is exceptionally challenging since serious and complex perioperative complications may occur in the perioperative period due to the high impact of stress response to the surgical trauma (2, 3). Complications include thrombosis of the coronary artery graft, postoperative bleeding, acute kidney injury (AKI), cerebrovascular accidents, arrhythmias, and death.

Studies on volume kinetics (4) provide an in-depth understanding of fluid distribution in different settings (5). Fluid management guidelines, meta-analyses and recommendations provide conceptual and practical advices for clinicians (6). However, the type of intravenous fluids and their combinations, as well as improved surgical techniques and enhanced recovery protocols are affecting outcomes (7). Thus, conflicting results from studies make it difficult to make conclusive recommendations based on the reported impact of different fluid management protocols on outcomes (8).

Edema is an independent predictor of organ hypoxia and severe outcomes (9). However, edema remains an unavoidable outcome of fluid therapy, especially in patients undergoing cardiac surgery with cardiopulmonary bypass (CPB). However, volume replacement in cardiac surgery patients remains controversial (10, 11). Given the complexity of fluid distribution in health (12) and in illness (4, 13), there is a need for continuous, clinically feasible, preferably noninvasive techniques for monitoring fluid accumulation with an ability to detect imminent deteriorations of hydration status during fluid loading (14). However, no technique has been proven clinically feasible for continuous assessment of hydration status (15).

Conceptually, the necessary fluid accumulation turns into adverse effect when it begins to overload circulation (hypervolemia) and/or tissues (edema), or underload circulation (hypovolemia) and/or tissues (dehydration) (5). These states are independent predictors of hypoperfusion, hypoxia and increased mortality (16). The threshold between beneficial and detrimental interstitial fluid accumulation is individual and hard to predict even in healthy individuals (17, 18).

Our previous research has addressed this issue by developing, on a theoretical basis, the HBS theory [see Supplementary Material 1] (19, 20), together with a transcapillary reflux model and a mini Volume Loading Test model (mVLT) (21–23) as tools for assessment and monitoring of transcapillary fluid shifts, volemia and hydration, and detecting imminent deteriorations based on plasma dilution response to fluid challenges using crystalloid solutions.

**Figure 1:**
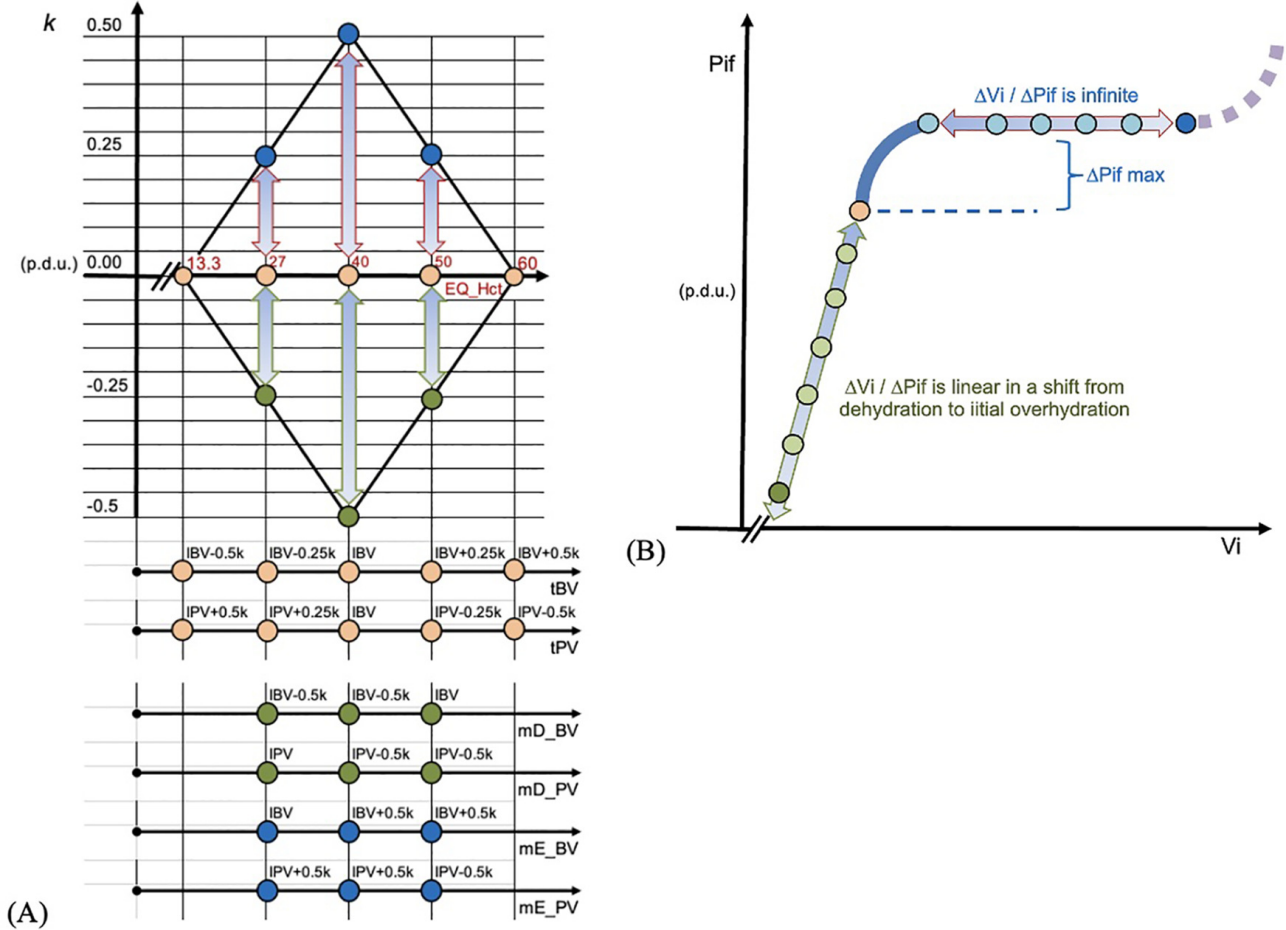
**(A)** EQ_Hct in our report refers to homeostatic target hematocrit (tHct) in HBS theory. EQ_Hct 13.3% refers to the Upper Hemodilution Limit (plasma dilution) in HBS theory. EQ_Hct 40% ITM is hematocrit at the Ideal Total Match, a unique combination of estimated ideal blood volume (IBV) and ideal plasma volume (IPV). EQ_Hct 50.0% refers to the Lower Hemodilution Limit (plasma concentration). The *rhombus* shape limits depict the red cell mass (RCM) dependent homeostatically *acceptable limits* of plasma dilution for a given EQ_Hct (where each EQ_Hct value corresponds to a unique red cell mass value); limits are based on the rule that neither blood volume, nor plasma volume cannot overcome the deviation from their estimated ideal values—IBV and IPV—by more than 0.5 *k*, where *k* is coefficient used in HBS math for estimating relevant data. mD_BV and mD_PV are values at the level of critical dehydration; mE_BV and mE_PV are values at the level of critical overhydration; Homoestatically safe upper plasma dilution limit is at EQ_Hct; to this state homeostasis strives to return soonest after this limit is overcome. **(B)** Ratio interstitial fluid volume (Vi) to interstitial fluid pressure (Pif) is interstitial fluid compliance. When it starts increasing (heavy blue curve), the maximal counterpressure to increased fluid filtration into tissues is reached; it facilitates reduction of interstitial fluid, e.g., via lymph. Theoretically, at that point homeostasis facilitates elimination of fluid from plasma so that excessive fluid is not shifting into tissues. It was assumed as happening during equilibration period of mVLT in our study. When the overwhelming fluid is entering tissues, e.g., capillary leak, compliance becomes infinite and fluids are accumulating easily in tissues. In extremes compliance starts decreasing and its life threatening edema condition.

The HBS theory (19) describes relationship between the red cell mass (RCM) and the amplitude of homeostatically acceptable large vessel plasma dilution fluctuation—an interval from imminent life-threatening plasma dehydration (plasma concentration) to plasma overdilution; schematically limits of plasma dilution fluctuation have a shape of rhombus (Figure 1). Theoretically, the homeostatic protection from overcoming the safe limits of plasma dilution is provided by fluid extravasation which is the net effect of elimination and shift into tissues, preferentially into derma. The transcapillary reflux model defines relationship between plasma dilution patterns and interstitial fluid compliance related states of tissue hydration [see Supplementary Material 2].

While the transcapillary reflux model and mVLT method has gained preliminary proof of concept in our previous trials, the HBS theory has not been tested although it was proposed more than two decades ago. This retrospective proof of concept study explored several previously untested aspects of HBS theory and transcapillary reflux model.

The present report evaluates the primary hypothesis of red cell mass (RCM) dependent amplitude of homeostatically acceptable limits of fluctuation in plasma dilution by exploring the correlation between RCM-specific equilibrated hematocrit (EQ_Hct) and amplitude of plasma dilution during perioperative mini Volume Loading Test (mVLT). We have retrospectively analyzed data collected in our previous RCTs—in elective total knee arthroplasty (TKA) and elective total hip arthroplasty (THA) patients. Perioperative mVLT protocols in TKA had a fixed number of fluid challenges (22, 23), while there was an individual number of challenges in THA trial because criteria for discontinuation of infusion was determined by automated decision support system (24).

The *primary endpoint* was relationship between the EQ_Hct and the PDA during mini Volume Loading Test (mVLT). The *secondary endpoint* was estimated fluid extravasation during 20 min equilibration period in mVLT [was used for testing secondary hypotheses (see Supplementary Material 2)].

## 2 Materials and methods

The material for the present study was derived from a database of perioperative intravenous infusion trials using mVLT method performed by our research team since 2009 (22–24). All studies were performed under the supervision of the first author. Trials were approved by the Vilnius regional biomedical research ethics committee, Lithuania (Ethical Committee numbers: 158200-9-071-22 and 158200-13-680-218) and conducted in accordance with the Declaration of Helsinki. Patient confidentiality was maintained through deidentification and secure data storage.

The mVLT protocol consisted of iso-osmotic crystalloid boluses each followed by a 5 min period without fluid. A 20 min period without fluids—*equilibration period*—was applied after last bolus. Invasive arterial hemoglobin concentration (aHb) and noninvasive SpHb were obtained before the 1st bolus, after 5 min period without fluid which followed each bolus, and after a 20 min equilibration period. Infusions were administered at a constant rate by an infusion pump. Blood (3 ml) for measurement of the aHb was withdrawn. Other details of the studies are reported elsewhere (22, 23).

The differences between the studies include the number of fluid challenges and the use of automated decision support system. Perioperative mVLT fluid protocols in TKA patients used fixed number of fluid challenges, while case dependent number was used in THA patients. 36 TKA patients received 3 boluses of 0.5 ml/kg of crystalloid (23), 48 TKA patients received 6 boluses of 0.25 ml/kg (22). All TKA patients mVLT protocol before induction of regional anesthesia and on the next morning. 34 THA patients received a case-specific number of boluses of 0.25 ml/kg determined by the automated decision support system within SCLIS before induction of regional anesthesia and in the evening of the day of surgery (unpublished results). The SCLIS is described in details elsewhere (25).

The study explored the HBS theory's claim that RCM dependent limits of homeostatically acceptable plasma dilution fluctuation—plasma dilution amplitude (PDA)—is changing bi-directionally from the plasma dilution at Ideal Total Match (ITM) Hct which maintains estimated ideal plasma volume (IPV) and ideal blood volume (IBV) (19, 20). Since fluids dilute RCM, the homeostatic target hematocrit (tHct) is used for referring to homeostatic target plasma dilution (tPD)—a state of *optimized plasma hydration*. The model defines a range of tHct as a range of “homeostatically acceptable” Hct values, e.g., if the ITM Hct is 40%, the model-estimated tHct range is from 13.3% to 60%; maximal PDA is at tHct 40%, and none at 13.3% and 60%. The tHct dependent PDA limits are schematically depicted as a rhombus shape (“Rhombus”) (Figure 1) [see Supplementary Material 1].

According to mVLT method, the plasma dilution after a 20 min equilibration period after last bolus is assumed as tPD in subjects without very significant pre-infusion deteriorations of hydration status and homeostasis. Thus, in this study we refer to Hct at this data point during mVLT as equilibrated hematocrit (EQ_Hct). We explored the PDA during perioperative mVLT with an aim to determine relationship between the PDA and the RCM-specific equilibrated hematocrit (EQ_Hct) which is consistent with optimized plasma dilution for a given RCM.

Analysis of plasma dilution trends was previously used for assessment of pre-fluid infusion hydration status by evaluating fluid extravasation in consecutive infusions (26–28). Thus, we used the difference in change of plasma dilution during equilibration period as indication of difference in fluid extravasation between mVLT protocols.

Although infused fluid dissolves in the blood, hemodilution has to be transformed into plasma dilution before it is used in calculations related to distribution of fluid, because it is the water in plasma that equilibrates with extravascular spaces. Our previous reports used estimation of plasma dilution trends from changes in hemoglobin where the baseline hemoglobin was before the first fluid bolus in mVLT. Mathematical methods were described elsewhere (21).

**Figure 2:**
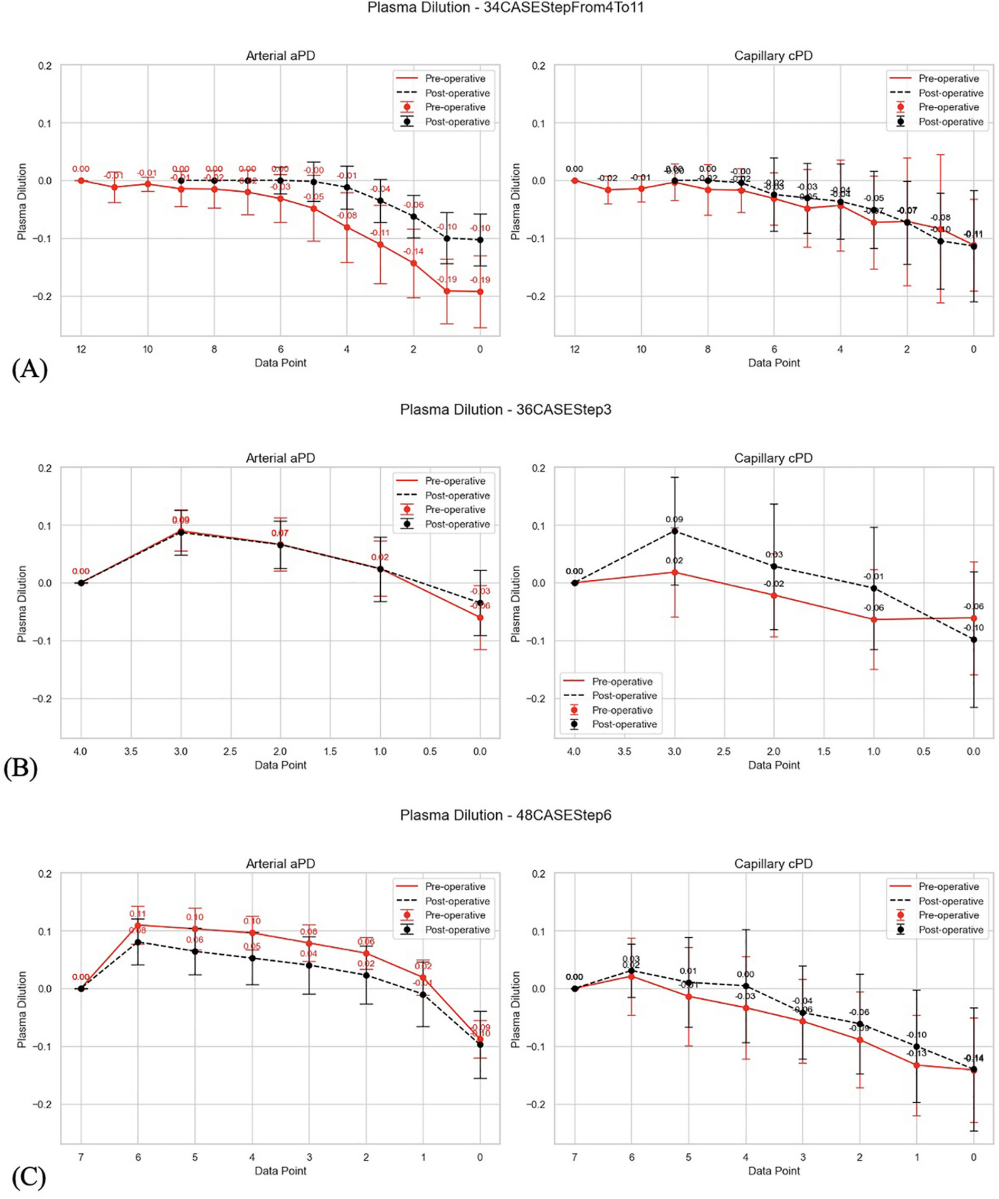
**(A)** Visualized differences between pre-operative and post-operative changes in arterial and capillary PD in perioperative mVLT sessions in 34 THA patients; reverse plasma dilution trends imply PD_EQ (first data point from the left) as baseline for estimates of PD during stepwise infusion; the PD which is before the first bolus (data point 0.0) is thus at the end of plasma concentration trend. **(B)** Visualized differences between pre-operative and post-operative changes in arterial and capillary PD in perioperative mVLT sessions in 36 TKA patients; reverse plasma dilution trends imply PD_EQ (first data point from the left) as baseline for estimates of PD during stepwise infusion; the PD which is before the first bolus (data point 0.0) is thus at the end of plasma concentration trend. **(C)** Visualized differences between pre-operative and post-operative changes in arterial and capillary PD in perioperative mVLT sessions in 48 TKA patients; reverse plasma dilution trends imply PD_EQ (first data point from the left) as baseline for estimates of PD during stepwise infusion; the PD which is before the first bolus (data point 0.0) is thus at the end of plasma concentration trend.

**Figure 3:**
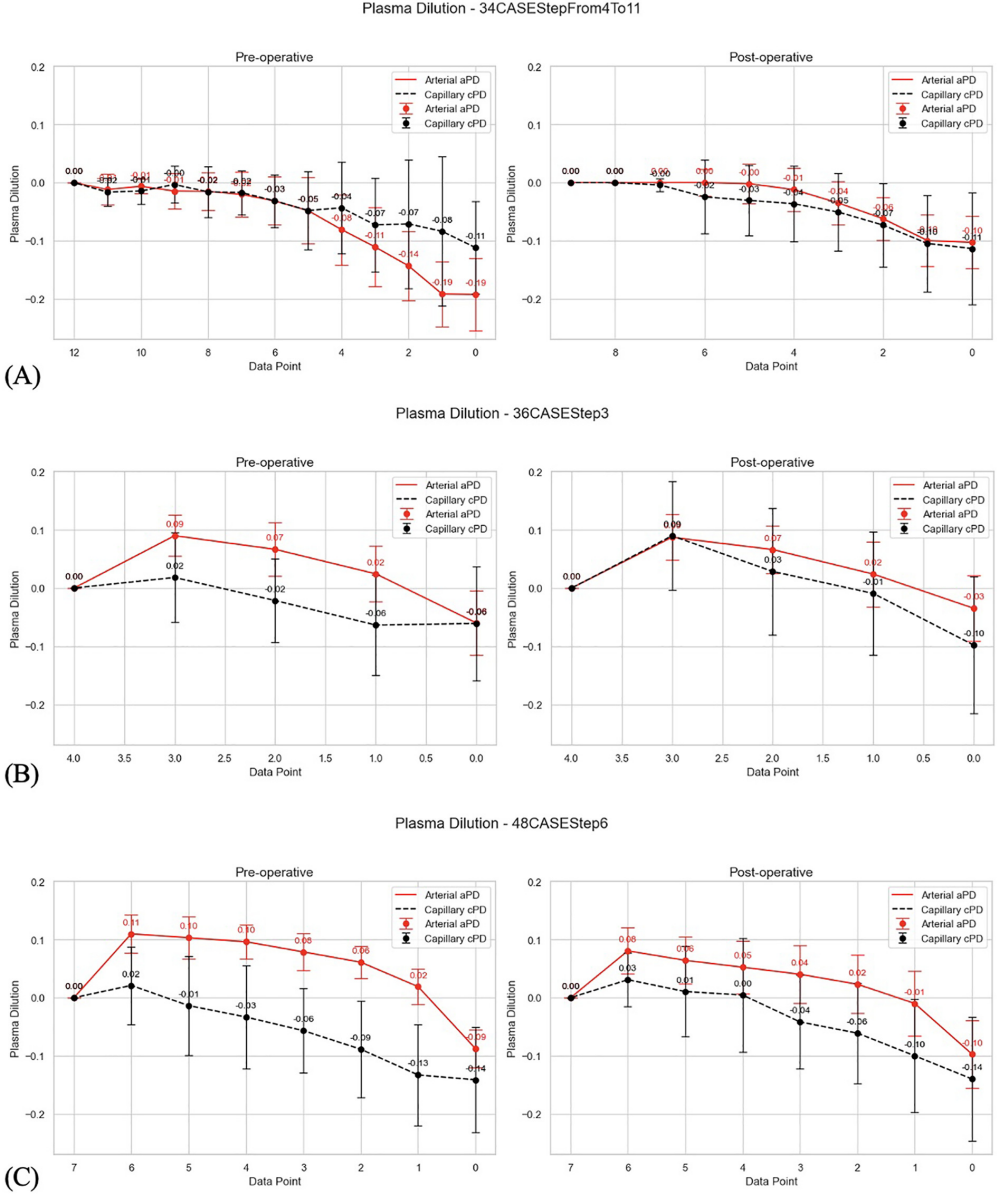
**(A)** Visualized differences between arterial and capillary PD. 34 THA patients; reverse plasma dilution trends imply PD_EQ (first data point from the left) as baseline for estimates of PD during stepwise infusion; the PD which is before the first bolus (data point 0.0) is thus at the end of plasma concentration trend. **(B)** Visualized differences between arterial and capillary PD.) 36 TKA patients; reverse plasma dilution trends imply PD_EQ (first data point from the left) as baseline for estimates of PD during stepwise infusion; the PD which is before the first bolus (data point 0.0) is thus at the end of plasma concentration trend. **(C)** Visualized differences between arterial and capillary PD. 48 TKA patients; reverse plasma dilution trends imply PD_EQ (first data point from the left) as baseline for estimates of PD during stepwise infusion; the PD which is before the first bolus (data point 0.0) is thus at the end of plasma concentration trend.

In this analysis we applied different approach to estimating plasma dilution. Hemoglobin concentration after 20 min equilibration period after last bolus in mVLT session was used as baseline (Figures 2, 3). Thus, a reverse trend in plasma dilution, which in fact is plasma-concentration trend, was used for analysis. This was used with an aim to have a standardized baseline plasma dilution because plasma dilution before the first bolus is obviously different in each subject [see Supplementary Material 2]. Since baseline hemoglobin was associated with EQ_Hct in plasma dilution calculations, the amplitude of *positive* plasma dilution values was used as an *indication of fluid extravasation* after the end of mVLT fluid protocol, and *negative* plasma dilution values were used as indication of pre-mVLT level of plasma (de)hydration. Invasive aHb was used for estimating aPD) and non-invasive SpHb (Radical 7; Masimo Corp., CA) was used for estimating capillary plasma dilution (cPD).

### 2.1 Data management and analysis

The *post hoc* analysis of 1,651 aPD, 1,645 cPD and 236 EQ_Hct estimates collected during 236 mVLT sessions in our previous RCTs (Tables 1, 2) was performed using Python 3.12.4, Tableau 2024.2.2, and SQL (Figures 2–10); [see Supplementary Material 5].

**Table 1:** Arterial data set summary statistics.

	Case	Bolus	Bolus steps	Hb	Hct	PD	HctEQ	HbEQ	aPDEQ
Count	1,651	1,651	1,651	1,649	1,651	1,651	236	236	236
Mean	20.86	3.29	5.52	111.7	0.34	0	0.34	111.2	0
Std	12.49	2.37	1.75	14.4	0.05	0.09	0.04	13.42	0
Min	1	0	3	73	0	−0.31	0.23	75	0
25%	10	1	4	101	0.31	−0.05	0.31	102.5	0
50%	21	3	6	112	0.34	0	0.34	111	0
75%	30	5	6	122	0.37	0.07	0.36	120	0
Max	48	12	11	154	0.47	0.19	0.44	145	0

**Table 2:** SpHb data set summary statistics.

	Case	Bolus	BolusSteps	cHb	cPD	HctEQ	cHbEQ	cPDEQ
Count	1,645	1,645	1,645	1,645	1,645	236	236	236
Mean	20.58	3.27	8.02	113.17	−0.04	0.33	109.24	0
Std	12.28	2.36	3.00	18.94	0.09	0.05	17.66	0
Min	1	0	4	48	−0.39	0.15	48	0
25%	10	1	7	100	−0.10	0.29	96	0
50%	21	3	7	112	−0.03	0.33	108	0
75%	29	5	12	125	0.00	0.37	122	0
Max	48	12	12	180	0.39	0.52	171	0

**Figure 4:**
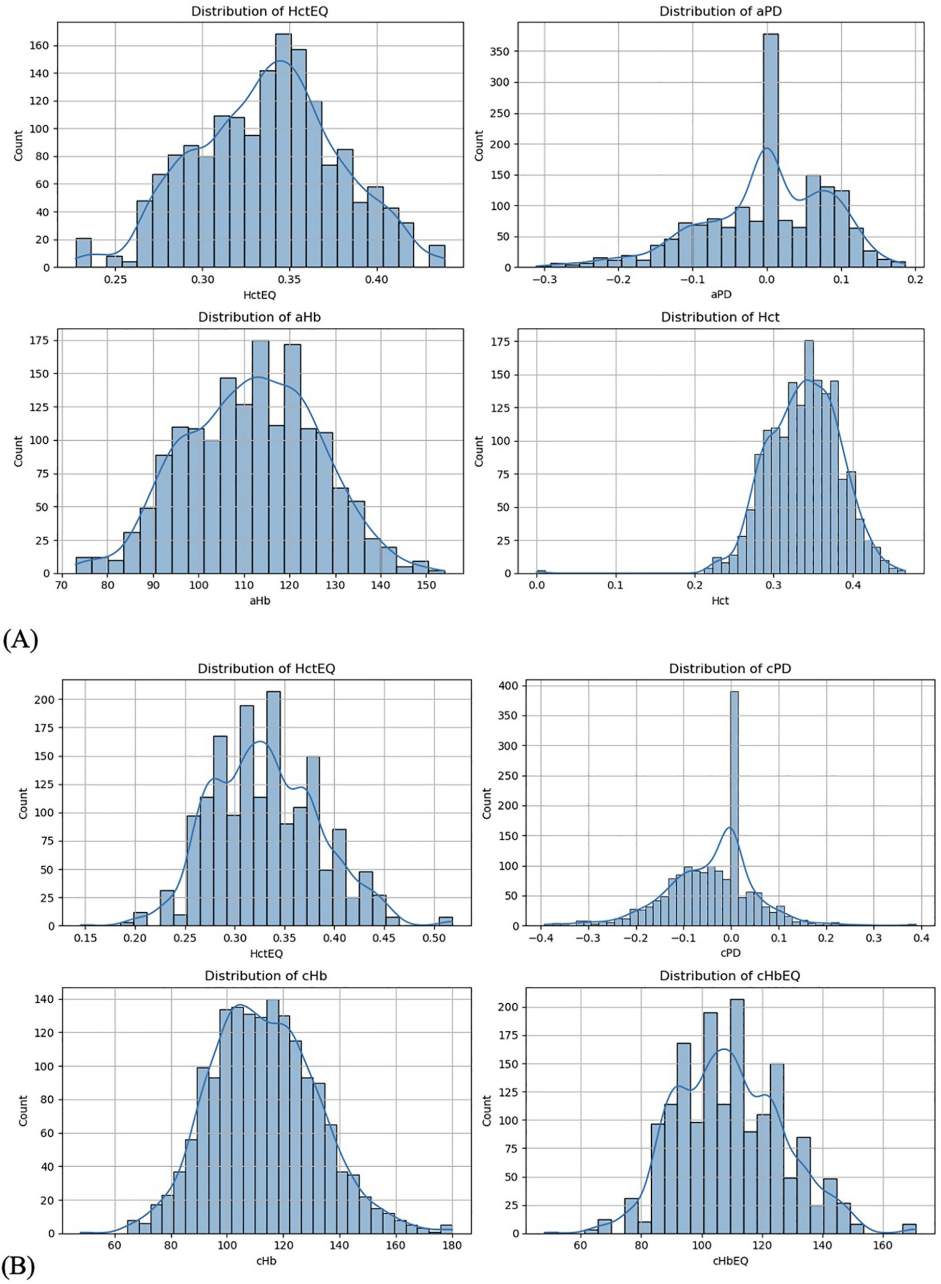
**(A)** Data distribution from arterial data set. **(B)** Data distribution from SpHb data set.

**Figure 5:**
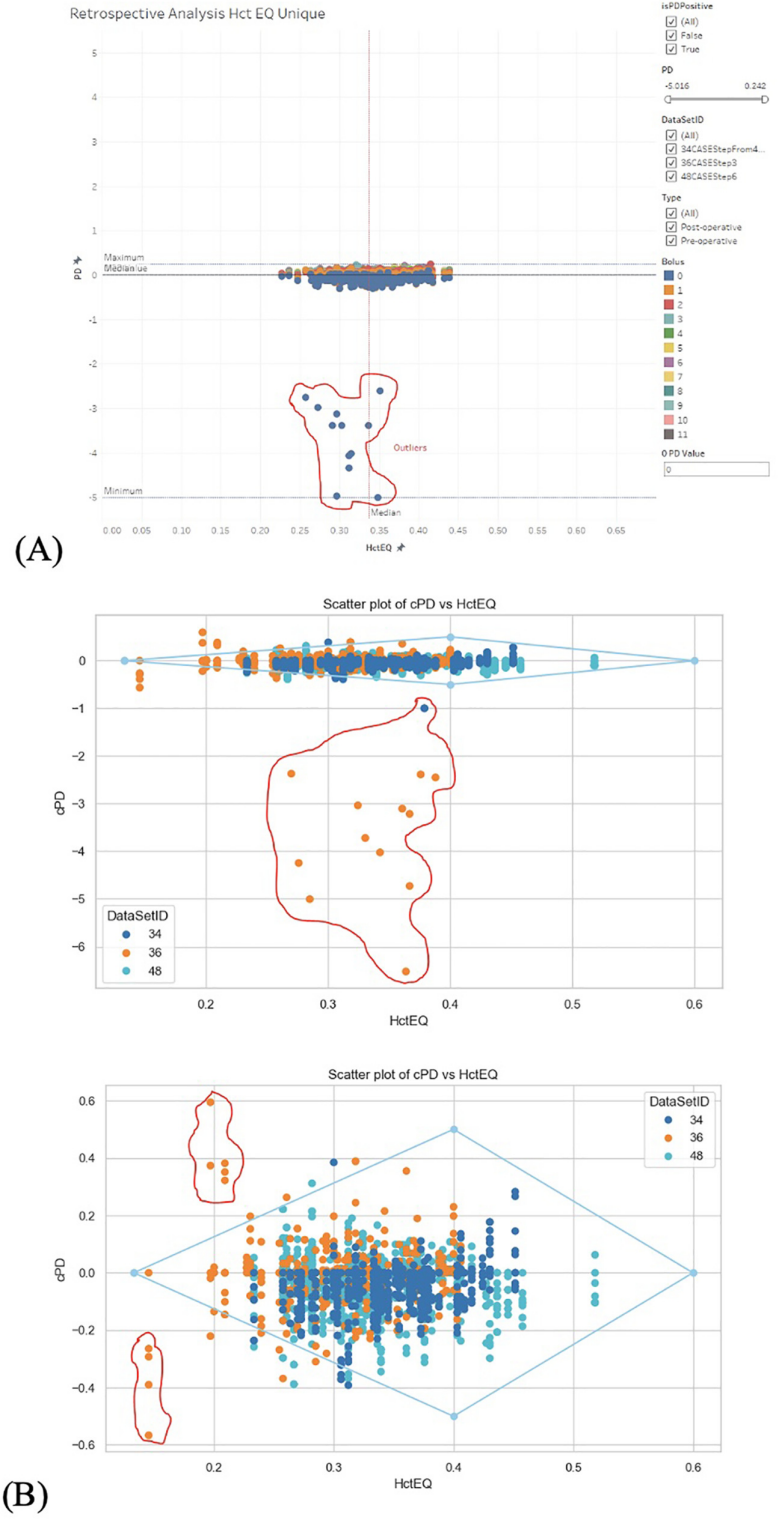
**(A)** Cleaning outliers in aPD_EQ data set. Step 1 and step 2. **(B)** Cleaning outliers in cPD_EQ data set. Step 1 and step 2.

**Figure 6:**
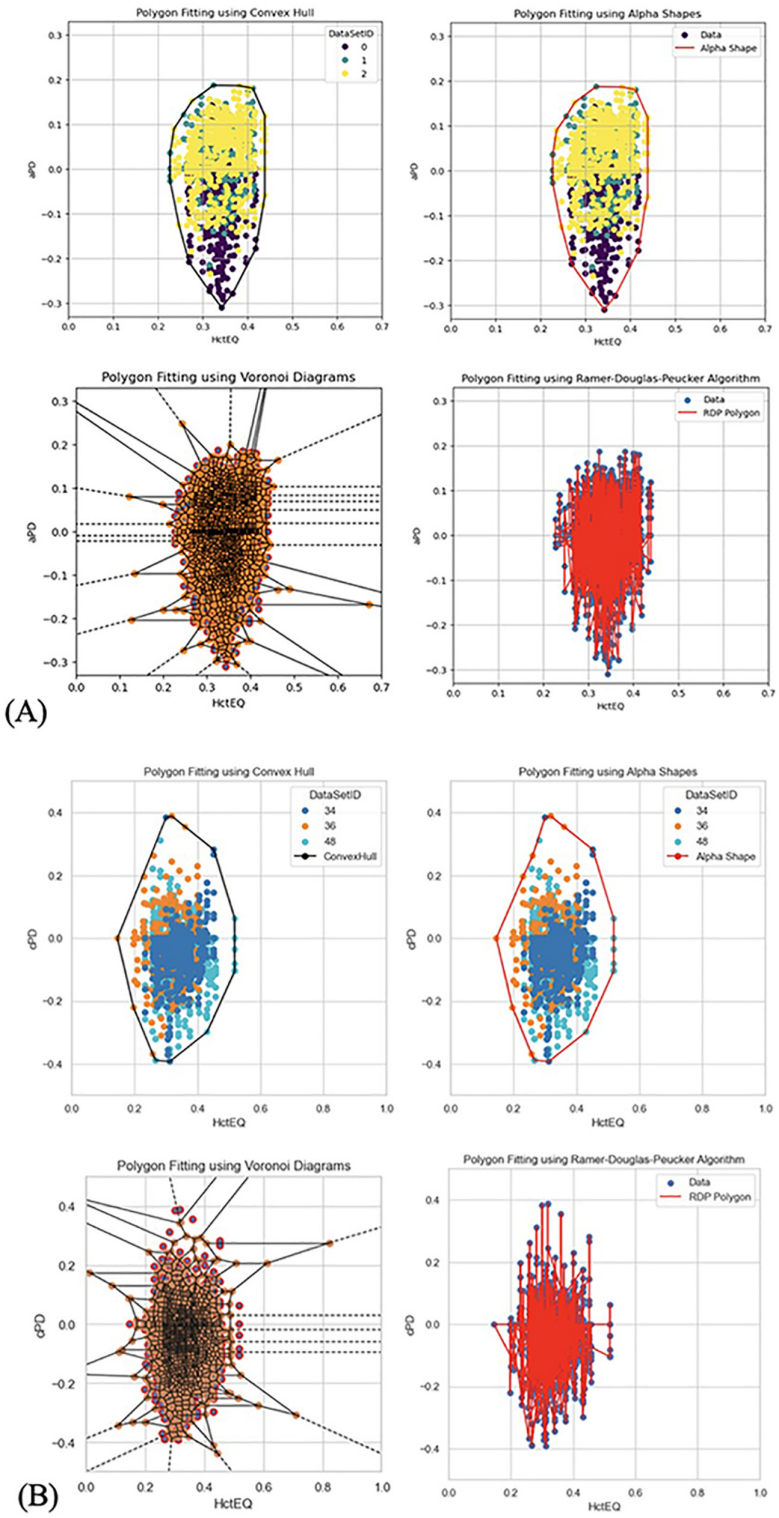
**(A)** Different methods to fit polygons in aPD_EQ data set. **(B)** Different methods to fit polygons in cPD_EQ data set.

**Figure 7:**
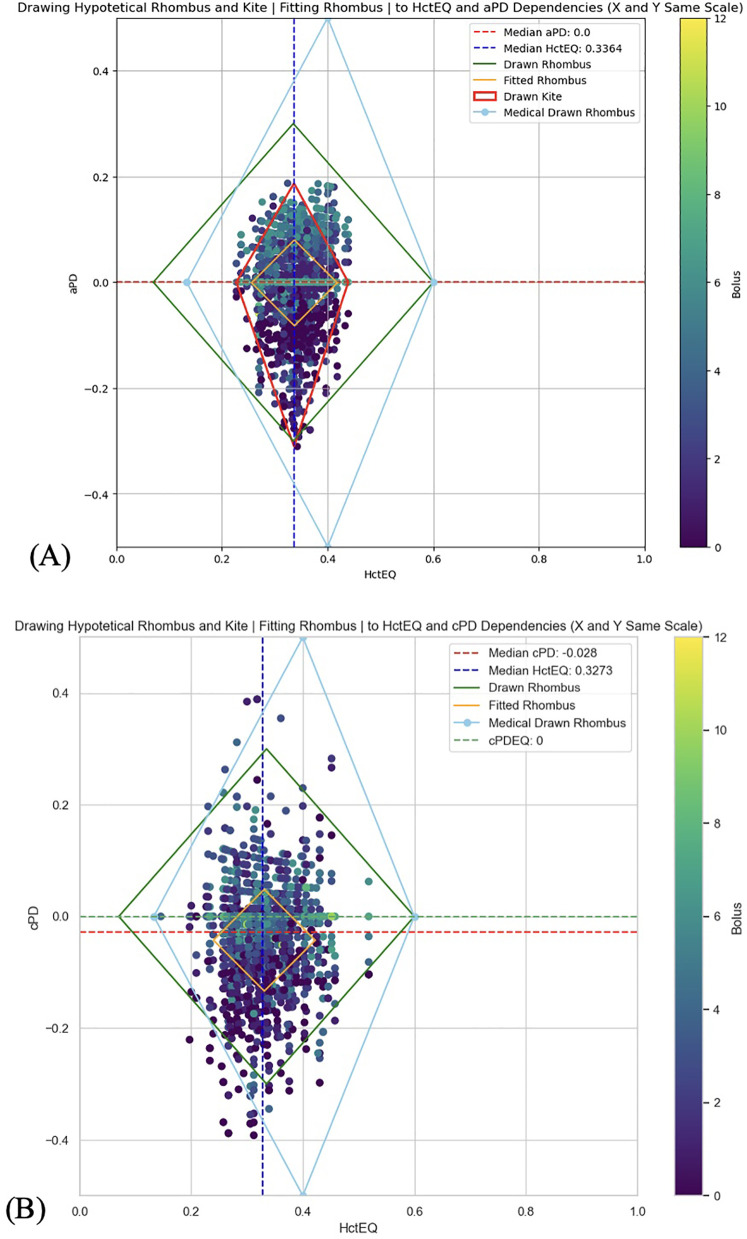
**(A)** A rhombus fitting method to assess the data distribution and its statistical relevance to follow rhombus shape (yellow line) for testing the theoretical red cell mass dependent limits of homeostatic plasma dilution. aPD_EQ data sets were fit to the HBS theory's “Rhombus” (light blue color; tHct range is from 13.3% to 60%. Maximal amplitude of PDA is at tHct 40%, and none at tHct 13.3% and 60%); (see Figure 1). **(B)** A rhombus fitting method to assess the data distribution and its statistical relevance to follow rhombus shape (yellow line) for testing the theoretical red cell mass dependent limits of homeostatic plasma dilution. cPD_EQ data sets were fit to the HBS theory's “Rhombus” (light blue color; tHct range is from 13.3% to 60%. Maximal amplitude of PDA is at tHct 40%, and none at tHct 13.3% and 60%); (see Figure 1).

**Figure 8:**
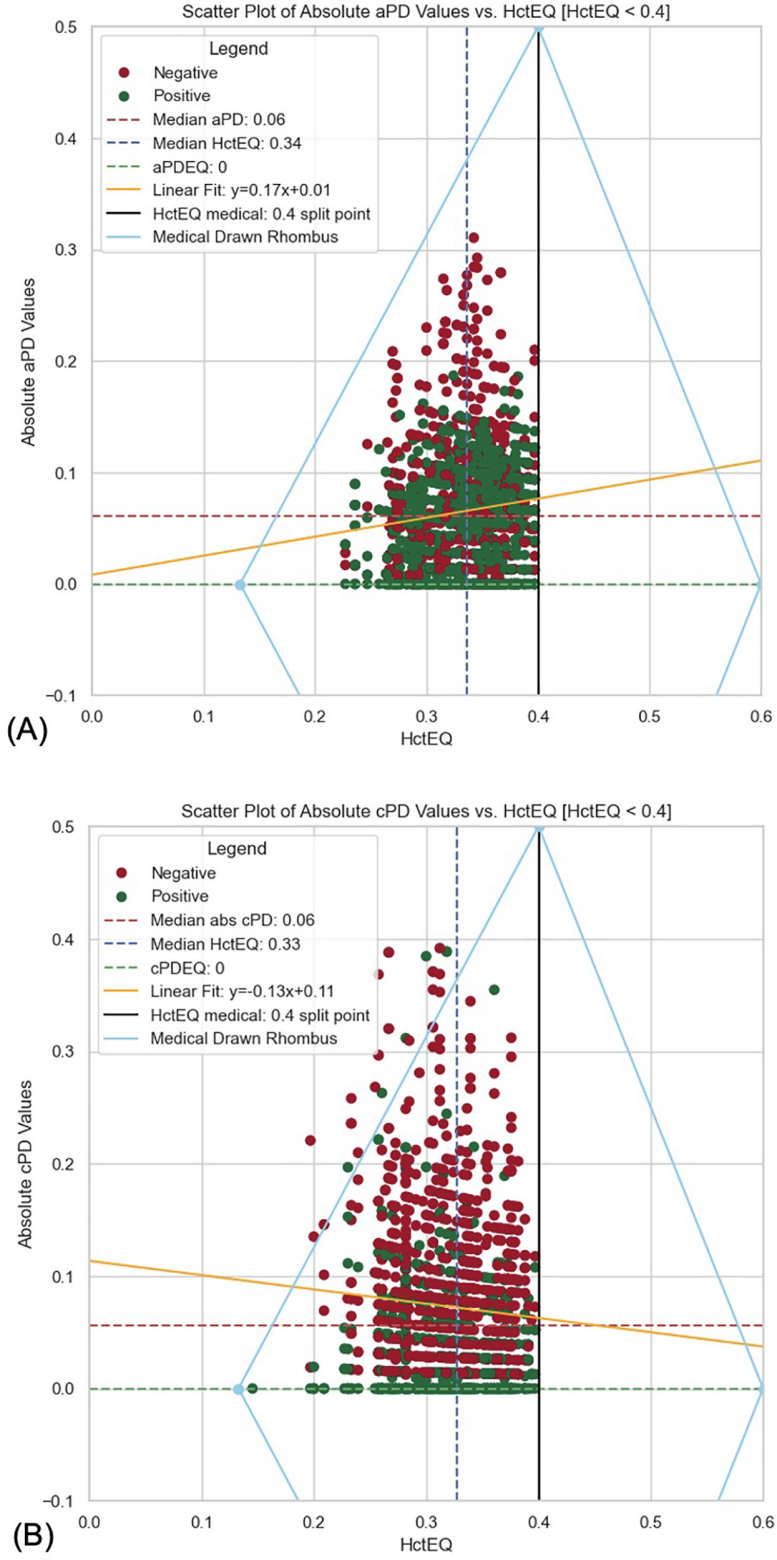
**(A)** Scatter plot of absolute aPD values vs. HctEQ [HctEQ < 0.4]. **(B)** Scatter plot of absolute cPD values vs. HctEQ [HctEQ < 0.4].

**Figure 9:**
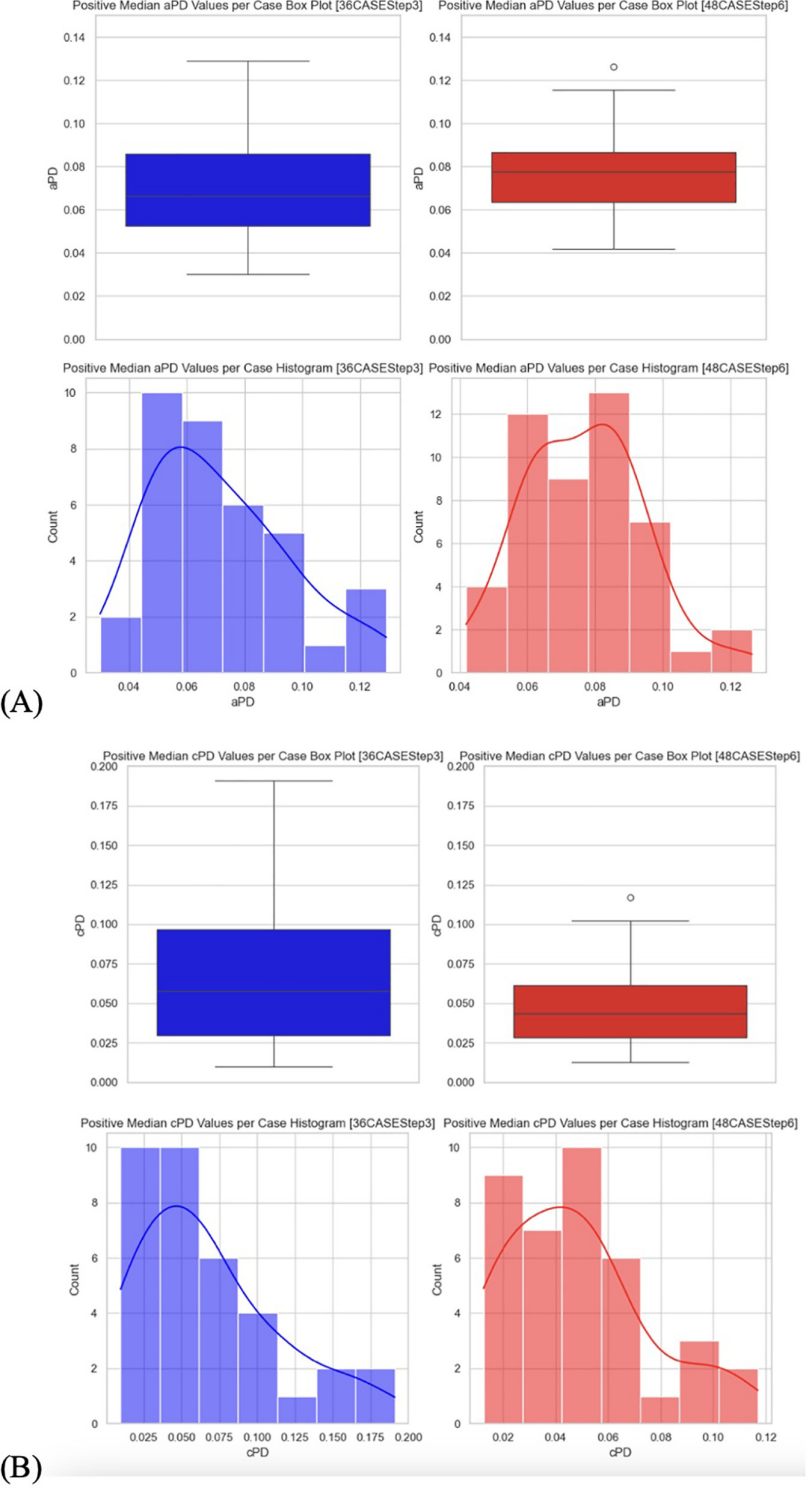
**(A)** Positive median PD values per case box plot and histograms. Positive median aPD values. **(B)** Positive median PD values per case box plot and histograms. Positive median cPD values.

**Figure 10:**
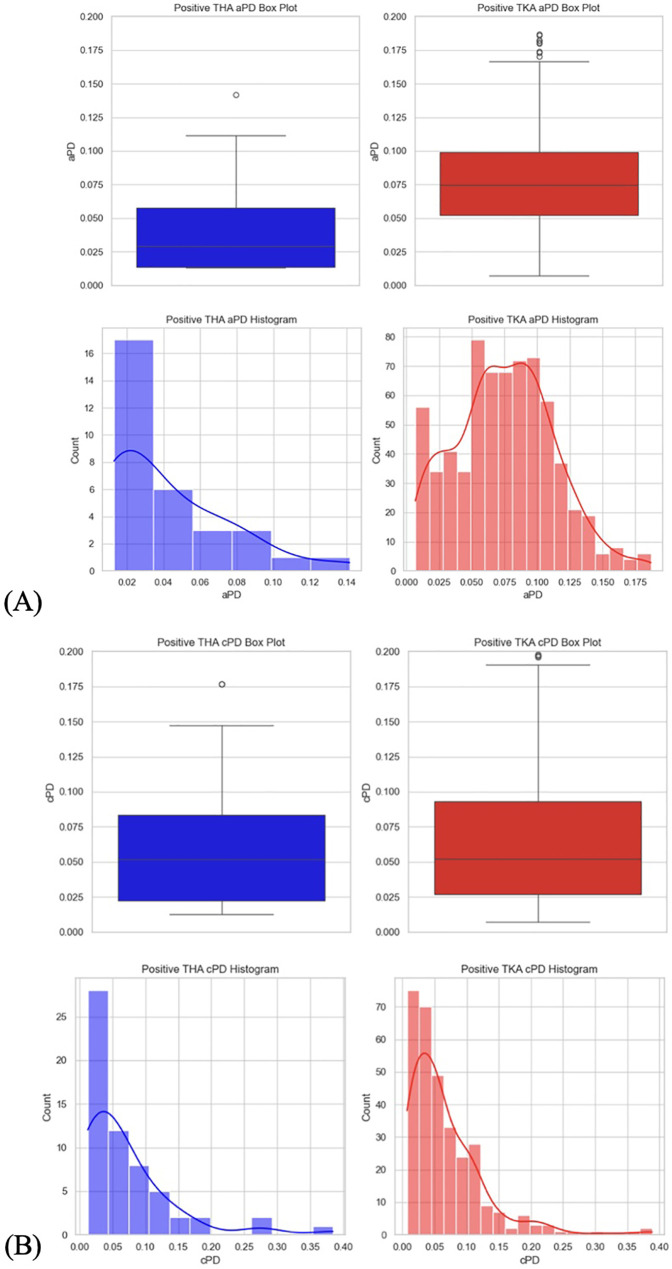
**(A)** Positive PD values per THA and TKA data sets box plot and histograms. Positive aPD values. **(B)** Positive PD values per THA and TKA data sets box plot and histograms. Positive cPD values.

#### 2.1.1 The analysis followed these steps

2.1.1.1 The primary hypothesis was that aPD and cPD during stepwise crystalloid infusion decrease with decreasing EQ_Hct when EQ_Hct is less than 0.4 (40%). Secondary hypotheses [see Supplementary Material 2] included: (a) different numbers of boluses with equal net volumes of crystalloid result in similar fluid extravasation during the equilibration period, and (b) an ACDSS suggested number of boluses induces less fluid extravasation compared to protocols with a fixed number of boluses.

2.1.1.2 Data distribution was visualized to understand the distribution of key numerical data columns (Figure 4). During data cleaning, rows where PD values were less than (−2) and appeared to be outliers or manual entry errors were removed (Figure 5). Outlier detection in raw data often relies on visual analysis methods since there is no baseline for comparison. Visual tools like scatter plots effectively reveal outliers by showing points isolated from clusters or patterns. Outliers are categorized into (58) point (global) outliers—individual points far from others; contextual outliers—points that deviate within specific conditions like time or space; and collective outliers—groups of points forming unusual clusters. Additionally, outlier values may be considered physiologically impossible based on the experimental context. Visual data analysis involved fitting polygons to analyze the shape and boundary of the data distribution between PD_EQ and Hct_EQ (Figure 6). We used geometric polygon fitting methods, such as Alpha Shapes, Convex Hulls, and Voronoi Diagrams, for analyzing the relationship between Plasma Dilution (PD) and Hematocrit Equivalent (HctEQ) to capture the true shape, spread, and boundaries of complex, non-linear data distributions without assuming a fixed functional form. These methods help identify clusters, outliers, and physiological limits, providing clear visual insight into how PD varies with HctEQ across different conditions. This approach supports model-free exploration of data patterns critical for understanding biological variability and limits.

2.1.1.3 A rhombus fitting method was employed to assess the relationship between Hct_EQ and PD_EQ in pre-operative and post-operative datasets, evaluating the data distribution and its statistical relevance to follow the rhombus shape (Figures 1, 7). The “Rhombus” testing involved fitting aPD_EQ and cPD_EQ data sets to the “Rhombus,” with tHct ranging from 13.3% to 60%. The maximal amplitude of PDA was at tHct 40%, with none at tHct 13.3% and 60% (Figure 4).

2.1.1.4 The aPD data sets were visualized to show changes in aPD from aPD_EQ (first data point from the left) to aPD at baseline (before the start of mVLT; data point 0.0) (Figures 2,3). Similarly, cPD data sets were visualized to show changes in cPD from cPD_EQ (first data point from the left) to cPD at baseline (before the start of mVLT; data point 0.0) (Figures 2,3).

2.1.1.5 Data where HctEQ was less than 0.4 were filtered for the primary hypothesis testing. Absolute values of PD were calculated, and positive and negative PD values separated the filtered data. Visual data analysis included plotting box plots and histograms for absolute PD values (Figure 8), followed by Spearman's rank correlation test between absolute PD and HctEQ values.

2.1.1.6 The secondary hypothesis testing (a) involved filtering the data sets 36CASEStep3 and 48CASEStep6. Data were grouped by Case and DataSetID, and median PD for positive values was calculated. Assumptions required for the test were checked using the Shapiro–Wilk test for normality. Visual data analysis included plotting box plots and histograms for each DataSetID (Figure 9). Welch's *t*-test was performed to compare the median positive PD values per Case in the two datasets, and mean values of median positive PD values per Case were calculated.

2.1.1.7 For testing the secondary hypothesis (b), data sets were filtered (tha_data = 34CASEStepFrom4To11; tka_data = 36CASEStep3 and 48CASEStep6). Positive PD values in THA and TKA filtered data sets were separated. The Shapiro–Wilk test for normality and Levene's test for homogeneity of variances were performed on THA and TKA-filtered data sets. Visual data analysis included plotting box plots and histograms for THA and TKA-filtered data sets (Figure 10). The Mann–Whitney *U* test was performed to compare the distributions of positive PD values in THA and TKA studies and mean positive PD values in THA and TKA studies were calculated.

## 3 Results

### 3.1 Invasive (arterial) data analysis

3.1.1 **The primary hypothesis** test result suggests a very weak positive relationship between HctEQ values and absolute aPD values (Figure 8), which is statistically significant. Spearman's correlation coefficient: 0.1025, *p*-value: 5.77 × 10^−05^; absolute aPD values decrease with decreasing EQ_Hct values when EQ_Hct < 0.4 (<40%).

3.1.2 **The secondary hypothesis (a)** test result suggests that there is no statistically significant difference between the positive median aPD of the 36 and 48 TKA patients' datasets (Figure 9A). Welch's *t*-statistic; *T*-statistic: −1.1081, *p*-value: 0.2721. [see Supplementary Material 2].

3.1.3 **The secondary hypothesis (b)** test result suggests that the mean amplitude of positive aPD values in 34 THA patients (0.0427) is significantly lower than in the pool of 36 and 48 TKA patients (0.0752) (Figure 10A). Mann–Whitney *U* test statistics: 5,327.5, *p*-value: 2.75 × 10^−06^. The results suggest that guiding fluid loading with SCLIS in the THA study achieved significantly lower positive cPD values compared to standardized number of boluses in the TKA studies.

### 3.2 Noninvasive (SpHb) data analysis

3.2.1 **The primary hypothesis** test result suggests a very weak negative correlation between HctEQ values and absolute cPD values, which is statistically significant. Spearman's correlation coefficient: 0.0640, *p*-value: 0.0149; this means that as HctEQ decreases, absolute cPD values tend to increase slightly, but the relationship is very weak.

3.2.2 **The secondary hypothesis (a)** test result suggests that there is no statistically significant difference between the positive median cPD of the 36 and 48 TKA patients' datasets (Figure 9B). Welch's *t*-statistic; *T*-statistic: 825.5, *p*-value: 0.0772. [see Supplementary Material 2].

3.2.3 **The secondary hypothesis (b)** test result suggests that the mean amplitude of positive aPD values in 34 THA patients (0.0694) is not significantly lower than in the pool of 36 and 48 TKA patients (0.0673) (Figure 10B). Mann–Whitney *U* test statistics: 8,974.5, *p*-value: 0.5371. The results suggest that guiding fluid loading with SCLIS in the THA study did not achieve significantly lower positive cPD values compared to a standardized number of boluses in the TKA studies.

## 4 Discussion

This retrospective proof-of-concept, study using our previously collected data has explored several aspects of HBS theory (19, 20) and transcapillary reflux model (22, 23), aiming to determine their feasibility for future implementation in devices for continuous noninvasive monitoring of hydration status in terms of fluid accumulation in tissues and/or circulation. The relevance of our work in the context of related issues in fluid therapy is briefly reviewed in Supplementary Material 3.

The results confirmed the *primary hypothesis* that the amplitude of both invasively and noninvasively estimated plasma dilution during stepwise crystalloid fluid loading decreases when the marker of optimized dilution—EQ_Hct—decreases, provided EQ_Hct <0.4 (i.e., <40%). This finding supports the HBS theory's claim that the amplitude of homeostatically “safe” fluctuations in plasma dilution depends on RCM and its inherent EQ_Hct value [see Supplementary Material 1].

A systematic review and meta-analysis (29) found that the decrease in large-vessel hemoglobin concentration (Hb) during hemodilution in both acutely and non-acutely ill patients is largest when the baseline Hb is >14 g/dl (29). This finding is consistent with our results and supports the main concept proposed by HBS theory: that optimized plasma dilution with EQ_Hct of approximately 40% (corresponding to Hb ∼12 g/dl) allows the widest margin for safe fluctuations in plasma dilution during fluid loading. Thus, a rehydrating infusion could have caused a decrease in Hb from 14 g/dl to 12 g/dl, a reduction also noted in the aforementioned study.

Fluid distribution studies using volume kinetic analysis in healthy volunteers who receiving rapid intravenous fluid loading have shown that high red blood cell and platelet counts are associated with greater peripheral accumulation of infused crystalloid solution (30). This is consistent with the HBS theory's concept that an amplitude of safe fluctuation in plasma dilution is decreasing bi-directionally from an EQ_Hct approximately 40% (corresponding to Hb 12 g/dl). Thus, the decreasing amplitude of fluctuation with both decreasing and increasing RCM correlates with a shift towards low and high red blood cell counts, respectively.

Theoretically, lower positive plasma dilution values (aPD and cPD) relative to plasma dilution at EQ_Hct indicate less plasma over-hydration during fluid loading and less extravasation of excessive fluid during equilibration period. Thus, we tested secondary hypotheses aimed at investigating differences between fluid protocols in terms of potential fluid overload during bolus administration by comparing the amplitude of plasma dilution reduction during a 20 min equilibration period. Our results confirmed the secondary hypothesis (a): that different numbers of boluses with equal net volumes of crystalloid result in similar fluid extravasation during the 20 min equilibration period following the last bolus. In TKA patients, three boluses of 5 ml/kg of Ringer's solution caused similar fluid extravasation as six boluses of 2.5 ml/kg. The amplitude of plasma dilution change during the equilibration period was used as an indicator of the amount of extravasation (Figures 2, 3B,C).

The results also confirmed the secondary hypothesis (b): that our prototype automated system, SCLIS-guided bolus number during perioperative mVLT sessions in THA patients, induced less fluid extravasation compared to the fixed number of boluses in TKA patients (Figures 2, 3A). The mean amplitude of positive aPD values in 34 THA patients was significantly lower than in the pooled group of 36 and 48 TKA patients. Finally, there is evidence that the pooled PD data from all three mVLT protocols are consistent with the theoretical “rhombus” shape of safe plasma dilution limits proposed by HBS theory (Figures 1, 5–8).

According to the HBS theory and the transcapillary reflux model, the dermis serves as the first line of defense against intravascular fluid overload beyond the homeostatic target plasma hydration level, which maintains EQ_Hct (as defined in this paper). This is primarily due to its role in regulating fluid balance and acting as a barrier. When there is an excess of fluid in the intravascular space, the dermis can absorb some of this excess, thereby minimizing the impact on the vascular system. While skeletal muscles are also capable of handling fluid shifts, they may respond later due to their greater vascularity and metabolic activity. The dermis, being more directly connected to the skin's surface and having a more immediate response capacity, plays a crucial role in the body's initial defense against fluid overload. As suggested by our results, overhydration was similar despite the two-fold difference in the number of fluid boluses. The same net fluid volume administered during the mVLT may not fully explain this finding, as slower infusion generally tends to result in less plasma overhydration. An increase in fluid elimination during consecutive infusions has been reported previously (26, 28). This has been explained by increased fluid extravasation, which represents the sum of fluid elimination and translocation into the interstitium, provided it remains lower than the increase in lymphatic influx. Thus, we propose that faster infusion (three boluses) would have resulted in even more pronounced overhydration compared to slower infusion (six smaller boluses), if it had not been counteracted by fluid extravasation once the limit of “safe” homeostatic plasma dilution (PD) was exceeded. Therefore, the positive aPD in both TKA groups was similar.

Although our results provide preliminary proof of concept, they should be interpreted with caution. The following paragraphs present study limitations and possible ways to mitigate some of them, absence of gold standard for hydration status assessment, and outlook of the discussed insights for evidence-based fluid management.

Firstly, a 20 min equilibration period was allowed for equilibration, during which no additional fluids are given. This period enabled the body to stabilize and distribute the previously administered fluid or medication before further interventions. However, the exact duration and necessity of such an equilibration period can vary based on the specific clinical context, the substances involved, and the patient's condition. Our choice of 20 min equilibration period was based findings from studies on volume kinetics (4) which reported that fluid distribution between compartments lasts for 20–30 min after the termination of a crystalloid infusion in both volunteers (31, 32) and surgical patients (33, 34). We have used a 20 min equilibration period in perioperative mVLTs for optimization of PD prior to obtain blood samples for measuring hemoglobin concentration which was used for estimating perioperative blood loss (35). The baseline hydration status can impact the volume of excess fluid accumulation during fixed number of bolus protocols, and thus fluid elimination during the 20 min equilibration period may be affected. However, the study design mitigated the impact of different baseline hydration statuses on intravascular fluid retention during infusion by starting fluid protocol at the same time in the morning (at 7:00 AM), with patients dehydrated after an overnight fast of approximately 10 h. Furthermore, the fluid protocol guided by the ACDSS demonstrated that individual bolus administration resulted in negligible fluid extravasation during the equilibration period, in contrast to the more significant and variable extravasation observed with fixed fluid volume protocols. Thus, the ACDSS-guided approach has the potential to reduce the risk of edema during fluid loading, for example, in conventional goal-directed fluid therapy guided by hemodynamic target parameters.

Secondly, we used the reduction in plasma dilution during the equilibration period as an indication of the net effect of fluid extravasation, which reflects the combined effects of elimination and fluid shift into tissues, since both influence plasma dilution. Although the net extravasation volume, as well as the volumes of fluid elimination and tissue shift, cannot be directly measured or reliably estimated using clinically practical methods, our methodology relies on an indirect index — the reduction in plasma dilution.

Thirdly, the concept of RCM dependent limits of “theoretically safe” PD fluctuation was tested based using the PD estimates in large vessels, and thus it is impossible to discriminate between the impact of two components of fluid extravasation—elimination and shift into extravascular compartment. Thus, changes in fluid accumulation in tissues—the hydration status—remain unclear. Meanwhile, deteriorations of hydration status are difficult to prevent because individual signs and symptoms have low sensitivity (36, 37), clinical diagnostic models are dependent on the skills of the provider (38), flow-related parameters are poor indicators of tissue hydration (39) and clinically practical continuous monitoring of interstitial fluid accumulation is not available (17).

Also, we retrospectively analysed data from previous RCTs that had different objectives compared to the present study, data set did not allow us to perform multivariate analysis. Thus, we plan future studies with appropriate design that will allow assessment of the impact of confounding factors.

There is no gold standard for hydration status assessment. Our method is unique in its approach to plasma volume and dilution assessment for estimating levels of hydration and detecting of imminent edema. Automated fluid and vasopressor administration systems that use continuous noninvasive monitoring of intravascular and extravascular fluid accumulation are among the expected developments in future technologies (14, 40). Among the few MW PPG-based prototypes for estimating hydration status that have been tested and reported was a wearable infrared spectroscopy device, termed the Sixty device. However, this device was unable to accurately assess changes in fluid status during or between dialysis sessions (15). A review of emerging technologies has emphasized the lack of a gold standard that could be used as a reference in dynamic settings, e.g., during fluid infusion (17). Thus, feasibility assessments of prototypes for measuring tissue hydration have not used reliable reference methods (15, 18, 41). A further discussion on similarities, as well as advantages and limitations of other methods is provided in [see Supplementary Material 4].

We have discussed insights that might be useful for developing innovative technologies and techniques. Aside from fluid consumption/infusion and changes in RCM, large vessel PD is affected by fluid extravasation, which is the sum of elimination and translocation into the interstitium. However, a change in large vessel PD is a poor indication of changes in extravascular fluid status. Thus, the development of continuous noninvasive techniques, most likely based on MW PPG, for estimating large vessel and capillary hemoglobin, as well as selectively measuring intravascular and extravascular water in the capillary beds of the dermis, is encouraged by our findings. This expectation is not very futuristic given the significant advance in current wearable MW PPG technologies (42). Our described techniques could be used for signal processing in MW PPG devices and provide clinically useful data for evidence-based fluid management and prevention of adverse effects of fluid therapy. The combination of known methods and new enabling technologies might open a path to a new era of clinical monitors.

## 5 Conclusion

In conclusion, this study provides preliminary yet promising evidence for the feasibility of noninvasive, continuous monitoring of hydration and fluid accumulation in perioperative patients using the HBS theory and transcapillary reflux model. Our results support the link between plasma dilution, RCM, and EQ_Hct, suggesting that safe dilution limits depend on RCM specific EQ_Hct. The finding that ACDSS-controlled infusion can minimise excessive infusion, highlights the potential for improved fluid management using automation in decision making and MW PPG technology for estimating plasma dilution. While further research is needed to refine these techniques, this study shows the role of dermis in counteracting fluid overload and offers a foundation for a more physiological approach to fluid administration in clinical settings.

## Data Availability

The datasets presented in this study can be found in online repositories. The names of the repository/repositories and accession number(s) can be found below: The datasets for this study can be found in the [figshare.com] links: [https://figshare.com/s/ef44952947159a4f0ba7]; [https://figshare.com/s/3e7bde8623d429a5b3a4]; [https://figshare.com/s/d83808136f0e0dfb49d5].
