# Supplementary material for: New insights in fluid monitoring for surgical patients. A concept study
Source: Front Med Technol. 2025 Jul 21;7:1619238. doi: 10.3389/fmedt.2025.1619238 (PMC12318955; doi:10.3389/fmedt.2025.1619238)
Supplement: Supplementary file 1 [file Table1.docx]

SUPPLEMENTAL MATERIAL 1

**HBS theory**

The HBS theory claims that limits of homeostatically acceptable fluctuations in hemodilution are changing bi-directionally from individual RCM value which is related to the state of estimated Ideal Total Match (ITM). It maintains the homeostatic target plasma dilution (tPD), estimated ideal plasma volume (IPV) and ideal blood volume (IBV). The ITM state has widest amplitude of so called maximal homeostatically “acceptable” PD, defined as a range from maximal dehydration (mD) to maximal overhydration (mE) (FIG. 1). However, homeostatically “safe” range is from mD to ITM. According to the theory, homeostasis prevents hemodilution over the tPV at ITM by all pathways of fluid extravasation. The safe range is an amplitude of “normal” fluctuation in plasma hydration. Exceeding these limits causes deterioration of intravascular and/or extravascular hydration up to life threatening dehydration or edema, and/or hypovolemia or hypervolemia.

The HBS theory proposed a *model* for estimating the RCM dependent tPD and amplitude of “safe” fluctuation based on these rules: (a) plasma dilution at maximal dehydration (PD_mD) is characterized by either maximal “safe” decrease in plasma volume below estimated IPV, or maximal “safe” decrease in blood volume below estimated IBV, (b) plasma dilution at maximal overhydration (PD_mE) is characterized by either maximal “acceptable” increase in plasma volume above estimated IPV, or maximal “acceptable” increase in blood volume above estimated IBV, and (c) there is no “acceptable” amplitude of plasma dilution fluctuations at “physiologically acceptable” lowest and highest tHct values since it would be a violation of rules (a) and (b). Conventional methods for estimating IBV and IPV apply.

The *model* implies that a set of IBV and IPV is maintained only once per “physiologically acceptable” range of RCM - it is only in tPD state at ITM. Since fluids dilute RCM, the model deploys target hematocrit (tHct) for defining tPD along the range of “homeostatically acceptable” hematocrit (Hct) values based on the relevant aspects of blood viscosity, rheology, coagulation and others. The HBS method provides mathematical model for estimating tPD, PD_mD and PD_mE based on the preferred ITM Hct value, e.g., when the ITM Hct is 40%, the model-estimated tHct range is from 13.3% to 60%. Maximal amplitude of PD fluctuations is at tHct 40%, and none at tHct 13.3% and 60%. The amplitudes are schematically depicted as a rhombus shape limits for tHct dependent mD and mE (FIG. 1).

Since tHct specific tPD is considered as the upper limit of “safe” plasma hydration, the HBS theory claims that homeostasis strives to return plaasma dilution to this limit during and after the overloading fluid entrance into circulation, e.g. IV fluid challenge. This concept is used in the Volume Loading Test (VLT) (1) and a transcapillary fluid reflux model; the mini VLT (mVLT) was proposed for better clinical feasibility compared to VLT(2).

In the present report, we analyzed plasma dilution during perioperative mVLT in major orthopedic surgery patients. We refer to plasma dilution and Hct after 20 min equilibration period without fluids after the last bolus as “equilibrated” - “equilibrated hematocrit” (EQ_Hct) and “equilibrated plasma dilution” (EQ_PD) for referring to tPD and tHct in the HBS model, accordingly. Since fluid loading induced PD from pre-infusion baseline is dependent on the pre-infusion hydration status, with an aim to evaluate fluid extravasation during equilibration period following the stepwise fluid infusion we used PD at EQ_Hct as baseline for estimating changes in PD during fluid protocols. It is a reverse trend of PD which is in fact a trend of plasma concentration.

1. Hahn RG, Andrijauskas A, Drobin D, Svensén C, Ivaskevicius J. A volume loading test for the detection of hypovolemia and dehydration. Medicina (Kaunas). 2008;44(12):953-9.

2. Andrijauskas A, Ivaškevičius J, Porvaneckas N, Stankevičius E, Svensen CH, Uvarovas V, et al. A mini volume loading test for indication of preoperative dehydration in surgical patients. Medicina. 2015;51(2):81-91.
