# Supplementary material for: New insights in fluid monitoring for surgical patients. A concept study
Source: Front Med Technol. 2025 Jul 21;7:1619238. doi: 10.3389/fmedt.2025.1619238 (PMC12318955; doi:10.3389/fmedt.2025.1619238)
Supplement: Supplementary file 2 [file Table2.docx]

SUPPLEMENTAL MATERIAL 2

**Transcapillary reflux model, implication in ACDSS, and secondary hypotheses**

Studies using volume kinetic analysis (1), as well as our studies (2-7) have found that continuous noninvasive hemoglobin (SpHb, Radical 7; Masimo Corp., CA) is not accurate for estimating large vessel plasma dilution. We assume that it is due to theoretical possibility that SpHb is sensitive to transcapillary fluid shifts since it is based on Multi-Wavelength (MW) Photoplethysmography (PPG) in capillaries of derma (7). Thus, in our previous research, the SpHb derived estimate of plasma dilution was referred to as capillary plasma dilution (cPD).

***Transcapillary reflux model***

With an objective to develop a method for indirect monitoring of fluid levels in tissues – the hydration status – in our previous research we explored the difference between invasively estimated plasma dilution – arterial (aPD) and/or venous (vPD) – and noninvasive cPD in healthy volunteers and elective total knee arthroplasty (TKA) patients. We found evidence that imminent edema can be detected during mVLT by analysing patterns of difference between aPD, vPD and cPD trends (2-4). The transcapillary fluid reflux model was developed as a background concept that defined relationship between the patterns plasma dilution and patterns of interstitial fluid compliance, the latter being inherent to specific clinically relevant states of hydration - dehydration, euhydration and overhydration (4).

To explore the model’s feasibility for providing advise to stop boluses when signs of imminent edema are detected, as well as assess its feasibility in an automated clinical decision support system (ACDSS), a study was performed to determine criteria for fluid responsiveness based on retrospective analysis of data from our previous RCT in TKA patients (7). We found that most sensitive and specific variables for assessment of fluid responsiveness and detection of imminent edema are arterio-capillary plasma dilution efficacy difference (acPED) and capillary plasma dilution efficacy (cPDE); the related math is described elsewhere (5). Most importantly, since acPED estimation requires both invasive aHb and non-invasive cHb, a major limitation of the method, we expllored the feasibility of solely noninvasive strategy. The grey zone approach was used to determine the cPDE value-intervals that can be used tas indication of fluid responsiveness, non-responsiveness, and inconclusive response, where non-responsiveness suggests imminent edema and is used to advise stopping boluses; its implication in mVLT algorithm was proposed (7).

***Feasibility of automated clinical decision support system (ACDSS)***

The ACDSS as part of our custom-built semi-closed-loop infusion system (SCLIS) was sucessfully tested *in silico* and in *Virtual Patient* studies (5-7) before SCLIS was used in our RCT for determining when to stop boluses during mVLT in 34 THA patients (unpublished results). Thus, patients received a case-specific number of boluses of 0.25 ml/kg crystalloid.

The aPD after bolusing which is estimated in respect to baseline before boluses is an indication of net intravascular *fluid accumulation* which is baseline fluid status dependent. Thus we used this approach in our previous studies on mVLT for comparing preoperative and postoperative hydration status, as well as exploring the dynamics of arterio-capillary, arterio-venous and veno-capillary plasma dilution difference trends during with an aim to define the criteria for detecting transcapillary fluid reflux as an indication of imminent edema.

***Secondary hypotheses***

In the present study our secondary objective was to assess and compare between fluid protocols *fluid elimination* during 20 min equilibration period after last bolus in mVLT was the reason why we used “reverse” plasma dilution trend where the baseline is a state after the fluid loading because the positive plasma dilution value is an indication of fluid elimination during the equilibration period. Hypothetically, the lesser the positive value, the lesser excess of fluid was infused. This ***secondary hypothesis (a)*** was used in the present study to explore if ACDSS guided number of boluses (in THA patients) has resulted in lesser excess infusion compared to fixed fluid protocols (in TKA patients).

The *secondary* hypothesis (a) was confirmed. The mean amplitude of positive aPD values in the 34 THA patients (0.0427) was significantly lower than in the pooled group of 36 and 48 TKA patients (0.0752) (Mann-Whitney U test: 5327.5, *p* < 0.001).

Additionally, with an aim to explore the efficacy of homeostatic forces to prevent intravascular fluid overload by eliminating excess fluids in maintaining the HBS theory defined homoestatic target plasma dilution, we tested the ***secondary hypothesis (b)*** that different number of boluses with different volumes but equal net volume of a crystalloid leads to similar fluid elimination during 20 min equilibration period (because homeostatic forces effectively eliminate excess fluid during the whole fluid session). The *secondary* hypothesis (b) was confirmed. There was no difference between the positive median aPD values of the 36 and 48 TKA patients (Welch’s t-statistic: -1.1081, *p* = 0.2721). This finding is also relevant for future studies using mVLT because it implies that various bolus volumes can be used with similar ability for detecting imminent edema during volume loading.

1. Hahn RG, Wuethrich PY, Zdolsek JH. Can perioperative hemodilution be monitored with non-invasive measurement of blood hemoglobin? BMC Anesthesiology. 2021;21(1):138.

2. Svensen CH, Stankevičius E, Broms J, Markevičius V, Andrijauskas A. Evaluation of hydration status calculated from differences in venous and capillary plasma dilution during stepwise crystalloid infusions: A randomized crossover study in healthy volunteers. Medicina (Kaunas). 2014;50(5):255-62.

3. Andrijauskas A, Svensen CH, Porvaneckas N, Šipylaitė J, Stankevičius E, Činčikas D, et al. A mini volume loading test (mVLT) using 2.5-mL kg−1 boluses of crystalloid for indication of perioperative changes in hydration status. Medicina. 2016;52(6):354-65.

4. Andrijauskas A, Ivaškevičius J, Porvaneckas N, Stankevičius E, Svensen CH, Uvarovas V, et al. A mini volume loading test for indication of preoperative dehydration in surgical patients. Medicina (Kaunas). 2015;51(2):81-91.

5. Andrijauskas A, Markevicius V, Navikas D, Porvaneckas N, Andriukaitis D, Stankevicius E, et al. In Vivo Testing of the Semi-Closed Loop Infusion System: the Preliminary Observations. Elektronika ir Elektrotechnika. 2015;21(1):28-32.

6. Markevicius V, Andrijauskas A, Navikas D, Dubauskiene N, Porvaneckas N, Stankevicius E, et al. In Silico Testing of the Semi-Closed Loop Infusion System with a New Simulator. Elektronika ir Elektrotechnika. 2014;20(9):19-24.

7. Markevicius V, Cincikas D, Porvaneckas N, Stankevicius E, Navikas D, Andriukaitis D, et al. Revised Evaluation of Hemodilution Response in the Semi-Closed Loop Infusion System. Elektronika ir Elektrotechnika. 2016;22(1):57-63.
