# Supplementary material for: New insights in fluid monitoring for surgical patients. A concept study
Source: Front Med Technol. 2025 Jul 21;7:1619238. doi: 10.3389/fmedt.2025.1619238 (PMC12318955; doi:10.3389/fmedt.2025.1619238)
Supplement: Supplementary file 3 [file Table3.docx]

SUPPLEMENTAL MATERIAL 3

**Problematics in fluid therapy and debate on the concept of third (fluid) space**

Optimization of blood volume and plasma dilution are among the main goals of fluid therapy. Conventionally, hemodynamic responsiveness is used as criteria for optimization of fluid status. However, there is poor relationship between intravascular and extravascular hydration status (1). Thus, indiscriminate correction of preload dependency with intravenous fluid bolus could lead to overhydration (2). Hemodynamic endpoints-guided optimization may cause deterioration of homeostasis due to excessive total body water, edema, dilutional coagulopathy and hemorrhage, hypoperfusion and hypoxia, and many other adverse effects of fluid therapy.

Fluid therapy is a highly debated topic (3). Understanding the pathophysiology of fluid distribution is essential. As a drug therapy, fluid therapy has a dose-response relationship (4) and requires careful and accurate assessment of fluid requirements, ensuring fluid levels in circulation and tissues are adequate (5, 6), and adverse effects are prevented (3). Since hundreds of publications by Prof. RG Hahn have provided in-depth understanding of fluid distribution in health and a variety of clinical settings, we will discuss only several aspects which are addressed in his recent works and which we think are most relevant to this paper.

Distribution of fluid from the plasma to the interstitial space may occur faster during general anesthesia as compared to the conscious state; the proposed explanation was that it ensures adequate nutritional flow to the cells despite decreased flow in the macrocirculation during general anesthesia (7). However, increased fluid shift into tissues does not cause reduction of plasma volume since its pronounced expansion occurs early during general anesthesia and is associated with accelerated lymphatic flow (8). Since such balance requires that lymph flux into circulation is higher than the shift of fluid into tissues, equilibration might need recruitment of fluid from remote extravascular fluid compartments to facilitate production of lymph. Theoretically, such remote source of fluid might be the elusive “third space”. However, other studies using kinetic models suggest that fluid tends to accumulate in the “third space” rather than being recruited during general anesthesia and surgery (9, 10).

The term “third fluid space” refers to the abnormal distribution of fluids to “non-anatomic” fluid compartment which may account for the increase in body weight for several days after major surgery (2). The recent kinetic analysis supported the probable existence of two interstitial compartments – one that equilibrates rapidly with the plasma and the other which equilibrates slowly, also referred to as a fast-exchange interstitial compartment and a slower-exchange compartment which might be referred to as the secluded “third space” (10). Fluid accumulation in extravascular space sequentially (11) recruits the fast-exchange interstitial fluid space and a remote "third space," the latter serving as an overflow reservoir (7). When more than 1 L was administered at a high rate, there was an expansion of a “third space”, which considerably extended the intravascular half-life (11). Meanwhile slower infusion might limit the “third-spacing” provided adequate diuretic response to plasma dilution. "Interstitial washdown" is an recently proposed term for edema-preventing mechanism that implies a greater redistribution of interstitial albumin into lymph whenever the capillary filtration is increased to the point of extravascular fluid overload (12).

The "third space" concept still causes much confusion. Theories and kinetic models for explanation of sequential fluid distribution between the fast and slow-exchange (“third space”) compartments were recently discussed (11). Although kinetic analysis of fluid volume shifts could identify two interstitial fluid compartments, it could not explain how they are connected to the bloodstream (13). Although they found that only the fast-exchange compartment is directly connected to the circulation, the anatomic characteristics of the “third space” was not proposed.

Given the complexity of processes and controversial research findings, we think that due to the similar interstitial fluid compliance of skin and skeletal muscles (14), as well as later involvement of muscles in fluid accumulation compared to involvement of skin, the skeletal muscles may be considered as the “third space”. They can accumulate several times more fluid compared to skin, and it may be stuck there much longer due to specific anatomy and physiology. Theoretically, monitoring interstitial fluid accumulation in skin, and stopping fluid infusion after detecting imminent fluid overload in derma may prevent clinically relevant detrimental fluid accumulation in the “third space”.

1. Dull RO, Hahn RG. Hypovolemia with peripheral edema: What is wrong? Critical Care. 2023;27(1):206.

2. Cihoric M, Kehlet H, Højlund J, Lauritsen ML, Kanstrup K, Foss NB. Perioperative changes in fluid distribution and haemodynamics in acute high-risk abdominal surgery. Critical Care. 2023;27(1):20.

3. Clinical Fluid Therapy in the Perioperative Setting. 2 ed. Cambridge: Cambridge University Press; 2016.

4. Payen D. Back to basic physiological questions and consideration of fluids as drugs. Br J Anaesth. 2014;113(5):732-3.

5. Chawla LS, Ince C, Chappell D, Gan TJ, Kellum JA, Mythen M, et al. Vascular content, tone, integrity, and haemodynamics for guiding fluid therapy: a conceptual approach. Br J Anaesth. 2014;113(5):748-55.

6. Hoste EA, Maitland K, Brudney CS, Mehta R, Vincent JL, Yates D, et al. Four phases of intravenous fluid therapy: a conceptual model. Br J Anaesth. 2014;113(5):740-7.

7. Hahn RG. Capillary Filtration of Plasma is Accelerated During General Anesthesia: A Secondary Population Volume Kinetic Analysis. J Clin Pharmacol. 2025.

8. Hahn RG. Accelerated lymph flow from infusion of crystalloid fluid during general anesthesia. BMC Anesthesiol. 2024;24(1):119.

9. Hahn RG. Fluid escapes to the “third space” during anesthesia, a commentary. Acta Anaesthesiologica Scandinavica. 2021;65(4):451-6.

10. Hahn RG, Dull RO. A Slow-Exchange Interstitial Fluid Compartment in Volunteers and Anesthetized Patients: Kinetic Analysis and Physiology. Anesthesia & Analgesia. 2024;139(2):339-48.

11. Hahn RG. Sequential recruitment of body fluid spaces for increasing volumes of crystalloid fluid. Frontiers in Physiology. 2024;15.

12. Hahn RG. Interstitial washdown during crystalloid fluid loading in graded hypovolemia –A retrospective analysis in volunteers. Clinical Hemorheology and Microcirculation. 2023;83(2):105-16.

13. Hahn RG. Evidence of serial connection between the plasma volume and two interstitial fluid compartments. Microvascular Research. 2024;151:104599.

14. Reed RK, Rubin K. Transcapillary exchange: role and importance of the interstitial fluid pressure and the extracellular matrix. Cardiovasc Res. 2010;87(2):211-7.
