# Supplementary material for: New insights in fluid monitoring for surgical patients. A concept study
Source: Front Med Technol. 2025 Jul 21;7:1619238. doi: 10.3389/fmedt.2025.1619238 (PMC12318955; doi:10.3389/fmedt.2025.1619238)
Supplement: Supplementary file 4 [file Table4.docx]

SUPPLEMENTAL MATERIAL 4

**State-of-the-art in using plasma dilution and other markers for assessment of hydration**

Change in plasma volume can be indicative of hydration status. In fluid experiments using volume kinetics and mVLT method, changes in plasma volume are estimated using hemoglobin and hematocrit levels. However, such measurements require controls for posture, arm position, skin temperature, and other factors. No advantages over osmolality (except hyponatremia detection for plasma sodium). Moreover, frequent blood draws is a major limitation while continuous noninvasive estimates of hemoglobin concentration are not a valid substitute. Isotopic tracers or specific biomarkers are used for the best accuracy in evaluating plasma dilution for assessing changes in plasma volume and whole body hydration status. However, they are analytically complex, expensive, invasive. Some studies utilize plasma protein (albumin) concentration changes to infer plasma dilution effects. However, albumin’s turnover via lymphatic loop reduces the specificity to changes in plasma volume and dilution.

A recent review (2020) (1) highlights the strengths and limitations of various hydration assessment methods, including complicated invasive methods (i.e. isotope dilution), to moderately invasive blood, urine and salivary variables, progressing to non-invasive metrics such as tear osmolality, body mass, bioimpedance analysis, and sensation of thirst. If methods such as salivary variables, urine colour, vital signs and sensation of thirst are utilised in isolation, great care must be taken due to their lack of sensitivity, reliability and/or accuracy. Fluid regulatory hormones can be affected by exercise and heat acclimation, requiring more complex analysis techniques. Detailed assessments such as neutron activation and stable isotope dilution analysis are highly accurate but expensive, with significant time delays due to data analysis providing little potential for immediate action. Since no single method is valid for all situations the recommended approach is to use their combinations (1, 2). While alternative variables such as hormonal and electrolyte concentration, bioimpedance and tear osmolality require further research to determine their validity and reliability before inclusion into any test battery.

Athletes present a unique subset of subjects for health monitoring research. Some endurance athletes consume a large volume of fluid that greatly exceeds the volume of fluid lost as sweat. Because sodium is lost in sweat, the outcomes are dilution of body fluids and reduced blood concentration of sodium (3). This phenomenon is appropriately named water intoxication. Not only does it degrade exercise performance, but severe cases may result in coma, pulmonary edema, cerebral edema, or death (4). Despite a substantial body of research, no best practice guidelines exist for the assessment of hydration in athletes (1, 5).

Although a few existing wearables measure athlete's performance, they are limited by a single function, rigidity, bulkiness, and required straps and adhesives. A recently (2024) introduced multi-sensor integrated wearable system (6) focuses on the assessment of dehydration in athletes through continuous monitoring of saliva and electrophysiological parameters. It utilizes a set of nanomembrane soft sensors and electronics, enabling wireless, real-time, continuous monitoring of saliva osmolality, skin temperature, and heart functions.

1. Barley OR, Chapman DW, Abbiss CR. Reviewing the current methods of assessing hydration in athletes. J Int Soc Sports Nutr. 2020;17(1):52.

2. Armstrong LE. Assessing hydration status: the elusive gold standard. J Am Coll Nutr. 2007;26(5 Suppl):575s-84s.

3. Montain SJ, Cheuvront SN, Sawka MN. Exercise associated hyponatraemia: quantitative analysis to understand the aetiology. Br J Sports Med. 2006;40(2):98-105; discussion 98-.

4. Convertino VA, Armstrong LE, Coyle EF, Mack GW, Sawka MN, Senay LC, Jr., et al. American College of Sports Medicine position stand. Exercise and fluid replacement. Med Sci Sports Exerc. 1996;28(1):i-vii.

5. Armstrong LE, Stearns RL, Huggins RA, Sekiguchi Y, Mershon AJ, Casa DJ. Reference Values for Hydration Biomarkers: Optimizing Athletic Performance and Recovery. Open Access J Sports Med. 2025;16:31-50.

6. Kim KR, Kang TW, Kim H, Lee YJ, Lee SH, Yi H, et al. All-in-One, Wireless, Multi-Sensor Integrated Athlete Health Monitor for Real-Time Continuous Detection of Dehydration and Physiological Stress. Adv Sci (Weinh). 2024;11(33):e2403238.
