# Supplementary material for: New insights in fluid monitoring for surgical patients. A concept study
Source: Front Med Technol. 2025 Jul 21;7:1619238. doi: 10.3389/fmedt.2025.1619238 (PMC12318955; doi:10.3389/fmedt.2025.1619238)
Supplement: Supplementary file 5 [file Table5.docx]

SUPPLEMENTAL MATERIAL 5

**Statistical analysis. Methods and algorithm flow**

We applied **advanced geometric fitting methods** to analyse the shape and boundary of the data distribution – we’ve fit a shape, not a relationship using “Polygonal Fitting Methods” or “Geometric Boundary Fitting” methods.

We used several methods for fitting polygons to data points, each with advantages and applications:

- The Convex Hull is the smallest convex polygon that can enclose all the data points. It helps find the boundary of a set of points.
- Alpha shapes are a generalization of the Convex Hull. They can capture the shape of a point cloud more accurately by allowing concavities.
- Voronoi diagrams partition the space into regions based on the distance to a set of points. The edges of the Voronoi cells can be used to form polygons.
- Ramer-Douglas-Peucker Algorithm. This algorithm simplifies a curve by reducing the number of points while preserving the overall shape.

**"Random curve fitting"** wouldn't be an accurate or appropriate term for these methods. Here's why:

**It's a deterministic algorithm**. Methods like **Convex Hull**, **Alpha Shapes**, and **Voronoi Diagrams** are **geometrically deterministic**. Given the same input data, they will always produce the same output. There's **nothing random** about how these algorithms fit shapes.

It is n**ot curve fitting in the traditional sense**. These techniques **do not attempt to model or predict** one variable as a function of another (like y=f(x), common in regression or curve fitting). Instead, they **analyze spatial structure** and **boundaries** of data point distributions.

**Convex Hulls**, **Alpha Shapes**, **Voronoi Diagrams**, and **Ramer-Douglas-Peucker** are **not related** to a **4th-order polynomial** or any polynomial fitting at all.

We are **bounding**, **describing**, or **analyzing the shape** of point distributions, not fitting a **curve** to **model or predicting** data behavior for visual data analysis.

Geometric polygon fitting methods, such as Alpha Shapes, Convex Hulls, and Voronoi Diagrams, are well-suited for analyzing the relationship between Plasma Dilution (PD) and Hematocrit Equivalent (HctEQ) because they capture the true shape, spread, and boundaries of complex, non-linear data distributions without assuming a fixed functional form. These methods help identify clusters, outliers, and physiological limits, providing clear visual insight into how PD varies with HctEQ across different conditions. This approach supports model-free exploration of data patterns critical for understanding biological variability.

For analyzing the relationship between PD and Hct, there are no known applications for us, but these methods are applicable in other biological contexts. Geometric polygon fitting methods application in Biological Contexts:

- <https://www.sciencedirect.com/science/article/abs/pii/S1877750311001001?via%3Dihub>
- <https://pmc.ncbi.nlm.nih.gov/articles/PMC6335759/>
- <https://pmc.ncbi.nlm.nih.gov/articles/PMC4828638/>

**Method/Algorithm Flow:**

1. Statistical Analysis
   1. Median Calculation. Medians for PD and HctEQ were calculated and rounded to four decimal places to be plotted as reference lines.
   2. Rhombus Fitting:
      1. Center Calculation. The center of the rhombus was determined by calculating the mean of HctEQ (x-axis) and PD (y-axis).
      2. Distance Calculation. Distances from the center to each data point were computed.
      3. Radius Calculation. The average distance (radius) was calculated.
      4. Vertices Calculation. Vertices of the rhombus were determined using the radius and predefined angles.
2. Rhombus Fitting Evaluation:
   1. The accuracy of the rhombus fit was evaluated using the Mean Squared Error (MSE) between the observed and predicted distances.
   2. The statistical relevance of the rhombus fit was assessed using the Chi-Square Test, comparing observed distances to expected values (radius).
3. Visualization. A scatter plot was generated to visualize the relationship between HctEQ and PD, with:
   1. Bolus colors data points.
   2. Reference lines for median PD and HctEQ.
   3. The fitted rhombus overlaid on the scatter plot.

To fit a rhombus to the data, we used the following formulas:

The center of the rhombus is the mean of the HctEQ and PD values.


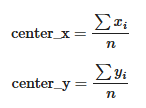


Equation 1

The Euclidean distance formula calculates the distance from each point to the center.


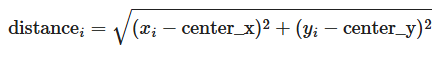


Equation 2

The average distance (radius) is the mean of the distances.


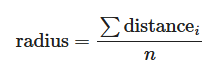


Equation 3

The angles for the vertices are defined as equally spaced points around a circle.


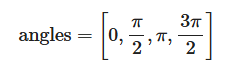


Equation 4

The vertices are calculated using the center, radius, and angles.


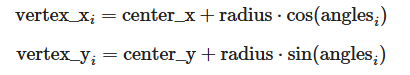


Equation 5

**Fit accuracy**

Mean Squared Error (MSE) of the rhombus fit: 0.0014. MSE measures the average squared difference between the observed and predicted values. A lower MSE indicates a better fit. In this case, the MSE is relatively low, suggesting that the rhombus model fits the data points closely.

Mean Absolute Error (MAE) of the rhombus fit: 0.0312. MAE measures the average absolute difference between the observed and predicted values. It provides a straightforward measure of fit accuracy in the same units as the data. The MAE value here indicates that, on average, the predicted distances deviate from the observed distances by approximately 0.0312 units.

Mean Absolute Percentage Error (MAPE) of the rhombus fit: 1.7155 or 172%. MAPE expresses the average absolute error as a percentage of the observed values. A MAPE of 172% indicates that the model's predictions are significantly off, with the average error being more than the actual values. This high MAPE suggests that while the model may fit the data well in absolute terms (as indicated by MSE and MAE), it struggles to accurately predict the values relative to their magnitude, which is visually apparent as data spread is very high.

R-squared of the rhombus fit: 0.4488. R-squared measures the proportion of variance in the observed data explained by the model. An R-squared value of 0.448 suggests that the rhombus model captures approximately 44.88% of the variability in the data. While this indicates a moderate fit, the data spread was expected to be very high.

Fitted rhombus values :

- Center of Rhombus (center_x): 0.337
- Center of Rhombus (center_y): -0.0013
- Distances from Center to Points: [0.1554 0.1445 0.0993 ... 0.1101 0.0946 0.02509]
- Average Distance (Radius): 0.0810
- Angles for Rhombus Vertices: [0. 1.5708 3.1416 4.7124]
- Vertices of Rhombus (vertices_x): [0.4181 0.3371 0.2560 0.3371 0.4181]
- Vertices of Rhombus (vertices_y): [-0.0013 0.07977 -0.0013 -0.08228 -0.0013]

**Statistical relevance of the rhombus fit**

The Chi-Square Statistic = 24.7192 measures the difference between the observed and expected values. A higher value indicates a more significant difference between the observed and expected values. The P-Value suggests the probability that the observed differences are due to chance. A P-value of 1.0 indicates no significant difference between the observed and expected values, meaning the observed data fits the expected model very well.

Given the P-value of 1.0, we conclude that the fitted rhombus is statistically relevant. The high P-value indicates that the observed distances from the center to the points are consistent with the expected distances based on the fitted rhombus model.

**Conclusion**

Overall, the rhombus model demonstrates a good fit to the data with low MSE and MAE values, indicating accurate predictions in absolute terms. However, the high MAPE value indicates significant relative errors, and the moderate R-squared value suggests that the model explains only part of the variance in the data. The Chi-Square Test yielded a high p-value, indicating no significant deviation from the expected distribution. This method effectively visualizes and evaluates the dataset's relationship between HctEQ and PD.
